# Postintroduction evolution contributes to the successful invasion of *Chromolaena odorata*

**DOI:** 10.1002/ece3.5979

**Published:** 2020-01-14

**Authors:** Weitao Li, Yulong Zheng, Likun Zhang, Yanbao Lei, Yangping Li, Zhiyong Liao, Zhongpei Li, Yulong Feng

**Affiliations:** ^1^ CAS Key Laboratory of Tropical Forest Ecology Xishuangbanna Tropical Botanical Garden Chinese Academy of Sciences Mengla China; ^2^ Institute of Soil Science Chinese Academy of Sciences Nanjing China; ^3^ University of Chinese Academy of Sciences Beijing China; ^4^ Center of Conservation Biology Core Botanical Gardens Chinese Academy of Sciences Mengla China; ^5^ Institute of Mountain Hazards and Environment Chinese Academy of Sciences Chengdu China; ^6^ Liaoning Key Laboratory for Biological Invasions and Global Changes Shenyang Agricultural University Shenyang China

**Keywords:** *Chromolaena odorata*, common garden experiment, EICA, founder effects, invasion

## Abstract

1. The evolution of increased competitive ability (EICA) hypothesis states that, when introduced in a novel habitat, invasive species may reallocate resources from costly quantitative defense mechanisms against enemies to dispersal and reproduction; meanwhile, the refinement of EICA suggests that concentrations of toxins used for qualitative defense against generalist herbivores may increase. Previous studies considered that only few genotypes were introduced to the new range, whereas most studies to test the EICA (or the refinement of EICA) hypotheses did not consider founder effects.
2. In this study, genetic and phenotypic data of *Chromolaena odorata* populations sampled across native and introduced ranges were combined to investigate the role of postintroduction evolution in the successful invasion of *C. odorata*.
3. Compared with native populations, the introduced populations exhibited lower levels of genetic diversity. Moreover, different founder effects events were interpreted as the main cause of the genetic structure observed in introduced ranges. Three Florida, two Trinidad, and two Puerto Rico populations may have been the sources of the invasive *C. odorata* in Asia.
4. When in free of competition conditions, *C. odorata* plants from introduced ranges perform better than those from native ranges at high nutrient supply but not at low nutrient level. The differences in performance due to competition were significantly greater for *C. odorata* plants from the native range than those from the introduced range at both nutrient levels. Moreover, the differences in performance by competition were significantly greater for putative source populations than for invasive populations.
5. Quantities of three types of secondary compounds in leaves of invasive *C. odorata* populations were significantly higher than those in putative source populations. These results provide more accurate evidence that the competitive ability of the introduced *C. odorata* is increased with postintroduction evolution.

## Graphical abstract

Three Florid, two Trinidad and two Puerto Rico populations may have been the sources of the invasive C. odorata in Asia. C. odorata increased resource investment into growth through post‐introduction evolution. Post‐introduction evolution enhances the competitive ability of C. odorata, and is essential for this species’ establishment and development in introduced ranges.

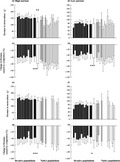

## 1 INTRODUCTION

The evolution of increased competitive ability (EICA) hypothesis suggests that invasive plants may reallocate resources from defense mechanisms into growth as a response to release from an enemy in their new range (Blossey & Notzold, 1995). Abela‐Hofbauerová and Münzbergová (2011) found that invasive *Cirsium arvense* in North America are larger in most size parameters than the native populations of the same species in Europe. However, most studies that evaluated this hypothesis have not differentiated specialist from generalist enemies, and, in introduced ranges, invasive plants may encounter generalist herbivores rather than total enemy release (Cano, Escarre, Vrieling, & Sans, 2009; Muller‐Scharer, Schaffner, & Steinger, 2004). Muller‐Scharer et al. (2004) refined the EICA hypothesis and proposed that, in introduced ranges, exotic plant species may adjust the allocation of resources from high‐cost quantitative defenses (i.e., resisting specialist herbivores) to growth and low‐cost qualitative defenses (i.e., resisting generalist herbivores). Nylund, Pereyra, Wood, Johannesson, and Pavia (2012) found that introduced *Fucus vesiculosus* increased the dosage of phlorotannins as a defense mechanism against generalist herbivores. The leaf total terpene contents in exotic plant species in Hawaii were 135% higher than that in native species, which facilitate them to resist the generalist herbivores from the introduced range, where specialist herbivores were scarce (Penuelas et al., 2010). Several studies have indicated that plant genotypes from introduced ranges had a more effective types of defenses than genotypes from native ranges (Blair & Wolfe, 2004; Joshi & Vrieling, 2005; Oduor, Kleunen, & Stift, 2017; Puritty, Mayfield, Azcarate, & Cleland, 2018; Turner, Hufbauer, & Rieseberg, 2014). Furthermore, Lin et al. (2015), based on features such as low root–shoot ratio, thin leaves, low leaf cell wall protein contents, and low leaf mass area, proposed that invasive *Jacobaea vulgaris* had poorer structural defense mechanisms than native genotypes.

Many plant secondary metabolites may act as defense mechanisms against herbivores and have allelopathic effects; if evolutionary mechanisms generate an increase in qualitative defenses against generalist herbivores, the allelopathic effect on indigenous plants may also be strengthened, eventually leading to the increase of the competitive ability of invasive species. Leaf extracts from the invasive *Chromolaena odorata* in China exerted stronger inhibitory effects on the germination of indigenous plants than the native populations of the same species from Mexico (Qin et al., 2013). Introduced *C. odorata* had higher resistance to three generalist herbivore species and higher tolerance to simulated herbivory (by shoot removal) than plants from native populations (Liao, Zheng, Lei, & Feng, 2014). Zheng et al. (2015) found that the concentration of odoratin (Eupatorium), a unique compound found in *C. odorata* with both allelopathic and defensive activities, in the introduced *Chromolaena odorata* was 2.4 times higher than that from the native range. The introduced population of *Taraxacum officinale* in the Chilean Andes produced more phenols and anthocyanins as a defensive response to herbivory than the native population in the French Alps (Gonzalez‐Teuber, Quiroz, Concha‐Bloomfield, & Cavieres, 2017).

Most common garden experiments that tested the EICA hypothesis did not include founder effects among the considered factors, which may have led to misleading conclusions, as source populations are only a fraction of the genotypes among native populations (Dlugosch & Parker, 2008). If the invasive populations are introduced from only one or a few native populations with stronger competitiveness, evidence supporting the EICA hypothesis would be found. However, source populations are weakly competitive, evidence contrary to the EICA hypothesis would be found. Both aforementioned situations could result from founder effects rather than postintroduction evolution. To exclude confounding founder effects, the difference between plants from invasive populations and the ones from their source populations should be compared (Williams & Fishman, 2014). For example, Sakata, Yamasaki, Isagi, and Ohgushi (2014) proposed that the features higher resistance, sexual reproduction, and asexual rhizome reproduction present in introduced populations of *Solidago altissima* resulted from a long history of pressure by *Corythucha marmorata* rather than from stochastic events such as genetic drift and founder effects.

*Chromolaena odorata* is a plant species native to North, Central, and South America, but is a noxious invasive perennial herb or subshrub throughout much of Asia, Oceania, and Africa. It was first introduced into India as an ornamental plant in the middle of the 19th century and has now become one of the most invasive species in southern China (Xie, Li, Gregg, & Dianmo, 2001). There are more than 200 arthropod enemies attacking *C. odorata* in its native range, and a quarter are specialists; however, some generalist herbivores are documented for *Chromolaena odorata* in invasive ranges, where specialists are absent (Zhang & Feng, 2007).

Although *C. odorata* has been introduced into Asia for nearly 100 years, the route of the spread of *C. odorata* throughout Asia is not well known. A study applying three DNA fragments and six pairs of microsatellite markers (SSRs) revealed that *C. odorata* in Asia originated from Trinidad and Tobago and adjacent areas in the West Indies (Yu, He, Zhao, & Li, 2014). However, Paterson and Zachariades (2013) indicated that the samples from Asia showed an affinity with samples from Trinidad, Florida, and Venezuela. Therefore, the sources of *C. odorata* in Asia could not be confirmed based on the existing researches (i.e., Paterson & Zachariades, 2013; Yu et al., 2014). Yu et al. (2014) indicated that the genotypes in Asia (introduced range) have strong competitive ability, which may facilitate the successful invasion of *C. odorata*. Therefore, it is reasonable to test the degree to which adaptation contributed to the higher competitive ability of *C. odorata* plants by comparing the invasive populations with the putative source populations.

During the invasion process, *C. odorata* strengthens its defense mechanisms against generalist herbivores (Liao et al., 2014) and enhances allelopathic effects (Qin et al., 2013). However, Liao et al. (2014) and Qin et al. (2013) did not consider the source of *C. odorata* and thus did not exclude founder effects. It is therefore necessary to confirm the source of *C. odorata* in native ranges and then compare invasive populations and source populations.

The hypotheses in this study are 1) the genetic diversity of *C. odorata* in native ranges is higher than that in introduced ranges; and 2) selective pressures in the introduced range cause an increase in the plant's growth rate and secondary compound production, which in turn increase its competitive ability. Evolution is predicted to increase the production of the secondary compounds active in defense and allelopathy, and to enhance growth traits.

## 2 MATERIALS AND METHODS

### 2.1 Plant materials

*Chromolaena odorata* weeds were collected in the species’ native regions in North America and the Caribbean and in the invasive ranges in Asia (Table S1). From each place (defined as a population), 10 plant seeds at least 20‐m intervals between any two plants were randomly selected and collected. A total of 10 and 12 geographical populations were collected from the invasive and native range, respectively. The seeds of various groups (populations) were seeded in the nursery bed.

### 2.2 Genetic analyses

Total genomic DNA was extracted from leaf tissues of *C. odorata* following the modified cetyltrimethylammonium bromide (CTAB) method described in Yu and Li (2011). In this study, 11 pairs of SSR primers were used to investigate the genetic diversity of 218 individuals from 10 introduced populations and 12 native populations (Table 1). The 11 PCR primers used are described in Table S1. The 20 μL PCR volume contained 1.5 μL of 10 × Buffer I, 1.0 µl of each primer (5 µmol/L), 0.3 µl of dNTP (10 mmol/L), 2.0 mmol/L of MgCl_2_, 0.1 µl of Taq polymerase (5 U/µL), and 15 ng of genomic DNA. PCR amplification was conducted using a Gene Amp 9,600 PCR system (ABI, USA). Amplification conditions were 10 min of denaturation at 95°C, followed by 35 to 40 cycles of 40 s at 94°C, 45 s at locus specific annealing temperature (Table S2), and 45 s at 72°C; and then a final extension step of 10 min at 72°C. Each forward primer was labeled with one of three fluorescent dyes (FAM, HEX, and TAMRA) for polymorphism analysis on a 3730xl DNA analyzer (ABI, USA) with internal lane Rox‐500 standards (Beijing Microread Genetics Co., Ltd, Beijing, China). PCR products with different sizes were multiplexed for detecting, and each mixture contained products of two to three primers labeled with different fluorescent dyes. The hierarchical partitioning of genetic variation within and among populations and regions was assessed by analysis of molecular variance (AMOVA; Armstrong & De Lange, 2005) based on the pairwise squared Euclidean distance among molecular loci using GenAlEx version 6.2 (Peakall and Smouse, Australian National University). Genetic relationships among native and invasive individuals were determined by constructing an UPGMA dendrogram using unweighted pairwise genetic distance matrices in POPGENE version 1.31 (Yeh & Boyle, 1997).

**Table 1:** Information on sample populations of *Chromolaena odorata*

Sample code	Country/region	G.P.S. coordinates	Elevation
Invasive populations			
JD	JingDong, Yunnan, China	24 º17'*N* 100 º50'E	1263
SM	SiMao, Yunnan, China	22º46'*N* 100º56'E	1380
ML	MengLun, Yunnan, China	21º56'*N* 101º15'E	544
SY	SanYa, Hainan, China	18º19'*N* 109 º12'E	23
WX	Vientiane, Laos	17º58'N102º37'E	170
BK	Central Thailand	14º25'N101º23'E	739
YNS	Southern Vietnam	11º20'N107º24'E	125
PH	Philippines	8 º 10'N124 º10'E	107
SL	Sri Lanka	7º11'N80º25'E	451
MY	Malaysia	2º22'N102º21'E	50
Native populations			
MAR	Florida, USA	27º06'N80º15'W	1 ~ 5
BRO	Florida, USA	26º08'N80º06'W	1 ~ 5
FAK	Florida, USA	25º52'N80º29'W	1 ~ 5
MD	Florida, USA	25º38'N80º20'W	1 ~ 5
CDV	Mexico	23º40'N99º11'W	600
CUB	Cuba	22º45'N82º50'W	565
MIC	Mexico	18º51'N103º37'W	950
PM	Puerto Rico	18º12'N67º06'W	103
PP	Puerto Rico	18º12'N67º06'W	103
COY	Mexico	16º44'N93º09'W	640
T2	Felicity, Trinidad & Tobago	10º31'N61º25'W	10
T1	Mamoral, Trinidad & Tobago	10º27'N61º17'W	63

### 2.3 Common garden pot experiment in China

A common garden experiment was conducted in China (introduced range) to explore the biogeographical differences in performance (biomass and height) of *C. odorata* between invasive and native populations (considering their source populations), and to test whether adaptive evolution contributes to the competitive ability of the invasive *C. odorata* by comparing the invasive and putative source populations. The common garden experiment located at the Xishuangbanna Tropical Botanical Garden (21°560′N, 101°150′E; 570 m elevation) of the Chinese Academy of Sciences, located in Mengla County, Yunnan Province, China. Annual average temperature is 21.7°C; mean temperatures of the hottest (July) and coolest (January) months are 25.3°C and 15.6°C, respectively. Average annual precipitation is 1557 mm with a dry period from November to April.

In December 2012, the above‐ground parts of *C. odorata* were cut to allow vegetative propagation by sprouting. In March 2013, sprouts of same sizes from 218 individual plants were selected and placed in sand beds. When the seedlings were 7 cm tall, similar‐sized vigorous seedlings were transplanted into 15 dm^3^ pots; ten individuals from each *C. odorata* population were planted one per pot. Twelve individuals of each of the ten invasive populations of *C. odorata* were transplanted into pots with each of the twelve native species, totaling 120 pots. A few EICA studies have performed studies of intraspecific competition (Felker‐Quinn, Schweitzer, & Bailey, 2013; Feng et al., 2009, 2011; Parker et al., 2013; Qin et al., 2013), which is important because intraspecific competition eliminates the potential confounding effects of using a heterospecific as a “phytometer.” Pots contained a mixture of 60% forest topsoil and 40% river sand. Topsoil was used as a natural supply of macro‐ and micronutrients, while river sand provided adequate drainage and facilitated the harvesting of fine roots (Liao, Zhang, Barclay, & Feng, 2013). All seedlings were initially grown in shade with 50% irradiance for 4 weeks to facilitate initial survival; after this period, they were grown in full sun.

Two types of nutrient treatments were set: low nutrient with one‐time fertilizer (in August 2013; fertilizer with 0.1 g N + 0.1 g P + 0.1 g K/Kg) and high nutrient with three‐time fertilizer (in June, July, and August 2013; fertilizer with 0.1 g N + 0.1 g P + 0.1 g K/Kg). The high and low nutrient treatments were harvested in September and December 2013, respectively. The entire plants (including roots) were oven‐dried at 60°C for 72 hr and weighed.

To evaluate relative competition intensity, the competitive response of each population was measured as the percentage change in performance (i.e., biomass) when grown with competition, and the formula was described by Weigelt and Jolliffe (2003):

(*P*_comp_ –*P*_single_)/*P*_single_ × 100,where *P*_single_ is plant performance when grown without competition and *P*_comp_ is plant performance when grown with competition. The competitive effect of each population was measured as the percentage change in the performance of its competitor. In this study, *P*_single_ was the average of all replicates per population per treatment and *P*_comp_ was the value of the individual replicate.

### 2.4 Secondary metabolite extraction and isolation

To detect the increase in production of qualitative defense factors, three types of secondary compounds were extracted and verified: high in defense capacity and allelopathy (4`,5,6,7‐tetramethoxyflavone and Acutellerin‐4`,6,7‐trimethy ether); high in defense capacity but low in allelopathy (*Isosakuranetin* and 3,5‐dihydroxy‐7,4`‐dimethoxyflavone); and low in defense capacity but high in allelopathy (dihydrokaempferol‐3‐methoxy ether and Kaempferide‐4`‐methoxy ether).

In April 2013, newly mature leaves of *C. odorata* were collected from five plants of each of the 12 native and 10 introduced populations (Table S1) grown in the common garden at the Xishuangbanna Tropical Botanical Garden, Chinese Academy of Sciences. All leaves were individually dried under room temperature and ground; then, 500 mg of powder was extracted using 50 ml of methanol for 24 hr. Above six chemicals were measured following the assay method described in Zheng et al. (2015), using ACQUITY ultra‐performance liquid chromatography (Waters Corp., Miller, MA, USA) equipped with a BECH C18 column (2.1 mm × 50 mm, 1.7 μm; Waters Corporation). The mobile phase included (A) pure water and (B) acetonitrile. The concentration of eluent B was changed from 10% to 60% by a linear gradient in 10 min. The flow rate of the eluent was 0.6 ml/min, the injection volume of the extract was 5 μL, and the column oven was set at 25°C. Conditions for mass spectrometric detection were as follows: Electrospray ionization (ESI) was performed in positive ion mode at 1.8 kV, ion source temperature was 350°C, solvent temperature was 550°C, sheath gas flow rate was 800 L/h, and auxiliary gas flow rate was 150 L/h. All data for 6 chemicals were collected using multiple reaction monitoring (Table S3). All measurements were obtained in the State Key Laboratory of Phytochemistry and Plant Resources in West China, Kunming Institute of Botany, Chinese Academy of Sciences.

### 2.5 Statistical analysis

Two‐way nested ANOVAs, with range and population nested within range as fixed factors, were used to estimate the significance of differences between plants from invasive and native populations of *C. odorata* at each nutrient concentration. The variations between plants from invasive and putative source populations of *C. odorata* at each nutrient concentration were determined using two‐way nested ANOVAs, with range and population nested within range as fixed factors. All analyses were conducted using SPSS 16.0 (SPSS Inc., Chicago, IL, USA).

## 3 RESULTS

### 3.1 Genetic diversity and structure

Genetic diversity in the invasive populations was significantly lower than in the native ones. Number of alleles, expected heterozygosity, and Shannon's information index (I) in native populations were, respectively, 1.8 (*F* = 13.15, *p* < .01), 1.1 (*F* = 7.66, *p* < .05), and 1.9 (*F* = 12.65, *p* < .01) times higher than those in invasive populations (Table 2).

**Table 2:** Diversity measures for polymorphic loci in 218 individuals of *Chromolaena odorata*

Locus	Native populations (*n* = 118)			I	Invasive populations (*n* = 100)			
	A	*Ho*	*He*		A	*Ho*	*He*	I
co77	5	0.03390	0.03368	0.1094	2	0.07000	0.06789	0.1517
co227	11	0.76316	0.77394	1.8159	4	1.00000	0.52705	0.8060
co250	11	0.61864	0.61576	1.2749	5	0.57000	0.43201	0.7186
co26	14	0.24138	0.53986	1.2702	5	0.04000	0.03970	0.1258
co115	12	0.30508	0.54064	1.1823	3	0.03000	0.02980	0.0874
co195	4	0.55932	0.43747	0.7771	2	0.97938	0.50238	0.6929
co65	19	0.44860	0.74560	2.0171	2	0.01000	0.01000	0.0315
co15	4	0.63559	0.45283	0.7359	2	0.94000	0.50070	0.6913
co56	4	0.31356	0.29005	0.5771	2	0.02041	0.02030	0.0569
co189	19	0.61864	0.86812	2.3040	8	0.33000	0.31688	0.7041
co50	10	0.73585	0.84400	1.9811	6	0.96939	0.53213	0.8490
Mean	**10.273**	0.47943	**0.55836**	**1.2768**	**3.727**	0.45083	**0.27080**	**0.4468**
St. Dev	5.623	0.23018	0.25220	0.6923	2.054	0.44592	0.23494	0.3457

Note

A, number of alleles; *He*, expected heterozygosity; *Ho*, observed heterozygosity; and I, Shannon's information index.

**Figure 1:**
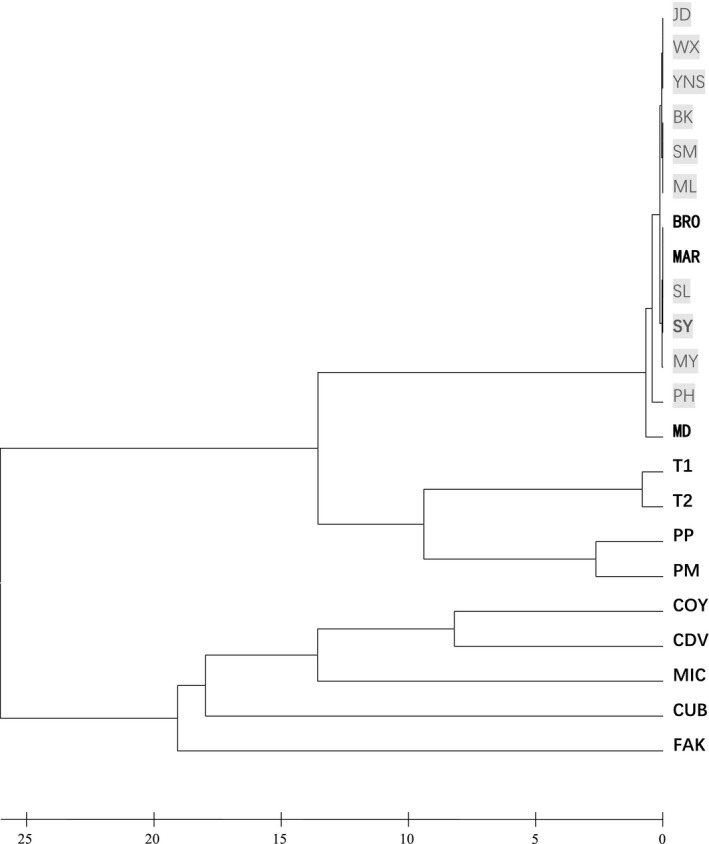
Cluster UPGMA of *Chromolaena odorata* plants from native and invasive ranges based on the data of microsatellite markers (SSR). JD: JingDong, Yunnan, China; WX: Vientiane, Laos; YNS: Southern Vietnam; BK: Central Thailand; SM: SiMao, Yunnan, China; ML: MengLun, Yunnan, China; BRO: Florida, USA; MAR: Florida, USA; SL: Sri Lanka; SY: SanYa, Hainan, China; MY: Malaysia; PH: Philippines; MD: Florida, USA; T1: Mamoral, Trinidad & Tobago; T2: Felicity, Trinidad & Tobago; PP: Puerto Rico; PM: Puerto Rico; COY: Mexico; CDV: Mexico; MIC: Mexico; CUB: Cuba; FAK: Florida, USA

These 22 populations consist of two separate groups. In one group, all invasive populations and three Florida populations (MD, MAR, and BRO) clustered together; in addition, two Trinidad populations (T1 and T2) and two Puerto Rico (PP and PM) populations were also close to them (Figure 1). The second group was formed by one Florida population (FAK), three Mexico populations (COY, CDV, and MIC), and the Cuba population (CUB).

### 3.2 Common garden pot experiment

**Figure 2:**
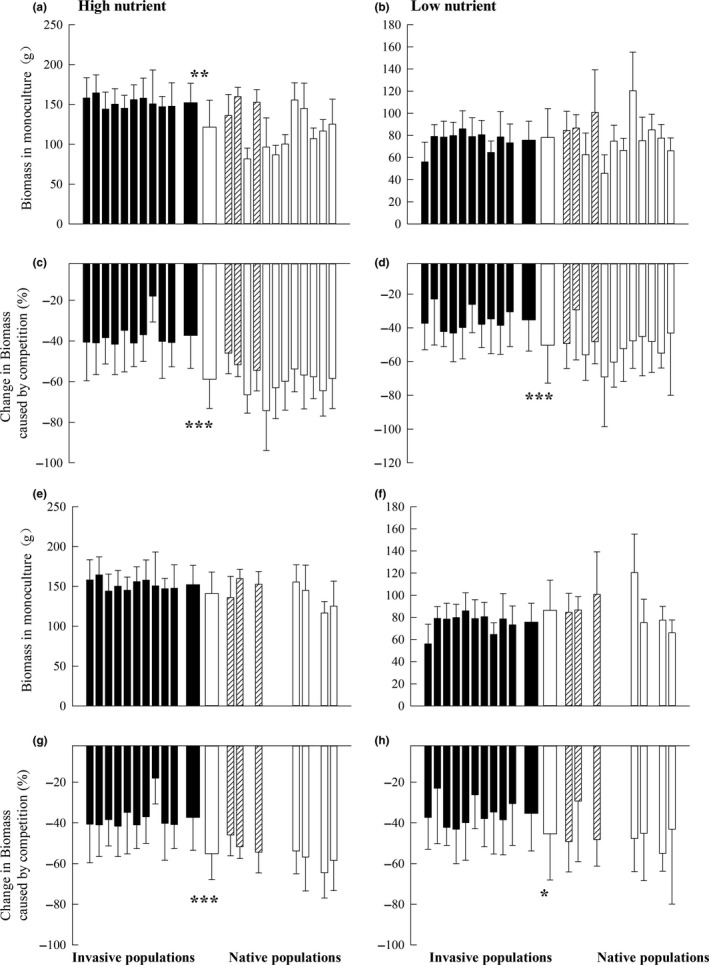
Comparisons between biomass of *Chromolaena odorata* plants from native and invasive populations, and invasive and putative source populations under high and low nutrient levels Populations grown in monoculture (a, b, e, f) and the respective changes influenced by competition (c, d, g, h). Panels a, c, e, and g represent plants grown at high nutrient level; panels b, d, f, and h represent plants grown at low nutrient level. Panels a, b, c, and d represent comparisons between invasive (*n* = 10) and native (*n* = 12) regions; panels e, f, g, and h represent comparisons between invasive (*n* = 10) and putative source (*n* = 7) regions. Striped columns represent Florida species. Narrow bars indicate mean + SE for each population (*n* = 10); central thick bars indicate mean + SE for each region (*n* = 10 for invasive; *n* = 12 for native). Significant differences between ranges according to one‐way nested ANOVAs: * = *p* < .05; ** = *p* < .01; *** = *p* < .001

**Figure 3:**
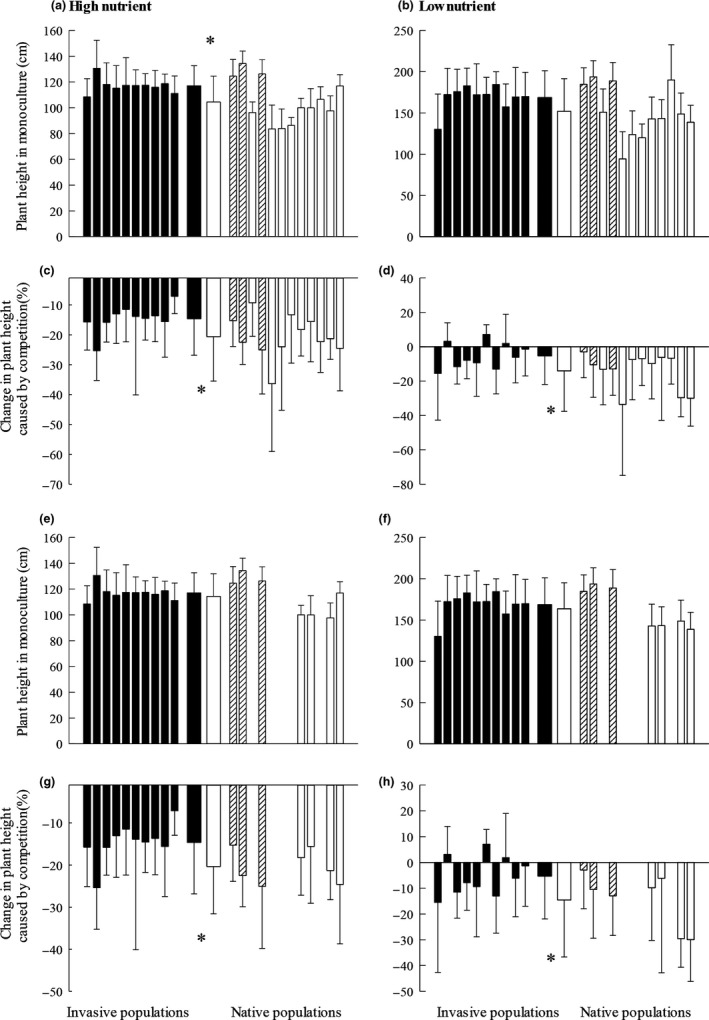
Comparisons between plant heights of *Chromolaena odorata* plants from native and invasive populations, and invasive and putative source populations. Populations grown in monoculture (a, b, e, f) and the respective changes caused by competition (c, d, g, h). Panels a, c, e, and g represent plants grown at high nutrient level, and panels b, d, f, and h represent plants grown at low nutrient level. Panels a, b, c, and d represent comparisons between invasive (*n* = 10) and native (*n* = 12) regions; panels e, f, g, and h represent comparisons between invasive (*n* = 10) and putative source (*n* = 7 for putative source) regions. Striped columns represent Florida species. Narrow bars indicate mean + SE for each population (*n* = 10); central thick bars indicate mean + SE for each region (*n* = 10 for invasive; *n* = 12 for native). Significant differences between ranges according to one‐way nested ANOVAs: * = *p* < .05

When grown without competition, total biomass and height (Figures 2a and 3a) of plants from the invasive range were larger than those of plants from the native range at high nutrient supply, but not at low nutrient level (Figures 2b and 3b). The relative competition intensity in total biomass and height of plants from the native range were significantly lower than those from the nonnative range (Figures 2c,d and 3c,d).

Regarding the comparisons conducted between *C. odorata* populations from invasive ranges and their putative source populations, when grown without competition, no significant differences in total biomass and plant height were observed between the two ranges at both nutrient levels (Figures 2e,f and 3e,f). Competition‐driven decreases in total biomass and plant height were significantly greater for *C. odorata* plants from the putative source populations than for those from the invasive ranges (Figures 2g,h and 3g,h).

### 3.3 Secondary metabolites differentiation

**Figure 4:**
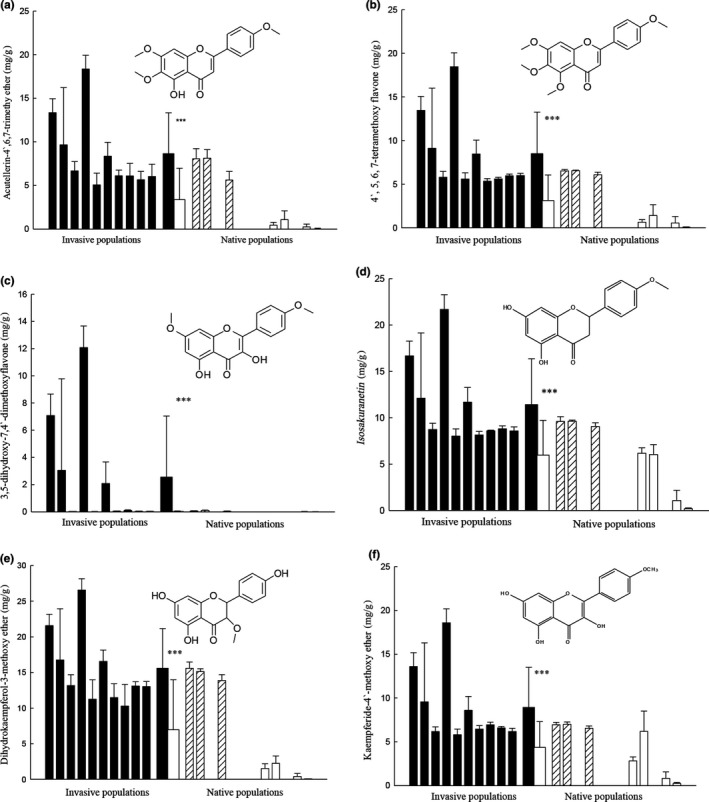
Comparison between secondary metabolite productions of *Chromolaena odorata* plants from invasive and putative source populations. Panels a, b, c, d, e, and f represent comparisons between introduced (*n* = 10) and putative source (*n* = 7) ranges. Striped columns represent Florida species. Narrow bars indicate mean + SE for each population (*n* = 10); central thick bars indicate mean + SE for each region (*n* = 10 for invasive; *n* = 7 for putative source). Significant differences between ranges according to one‐way nested ANOVAs: *** = *p* < .001

Concentrations of the three extracted and verified types of secondary compounds (4`,5,6,7‐tetramethoxyflavone and Acutellerin‐4`,6,7‐trimethy ether; *Isosakuranetin* and 3,5‐dihydroxy‐7,4`‐dimethoxyflavone; and dihydrokaempferol‐3‐methoxy ether and Kaempferide‐4`‐methoxy ether) in leaves of *C. odorata* plants in nonnative ranges were consistently and significantly higher than that in putative source populations (Figure 4).

## 4 DISCUSSION

The level of genetic diversity of *C. odorata* plants throughout Asia is significantly lower than that in native populations; similar results were reported by Ye, Mu, Cao, and Ge (2004) and Yu et al. (2014). In introduced ranges, populations of an invader often originate from only few individuals from the native range, and the invasion into a new territory is associated with frequent founder effects, which potentially lead to a decrease in population‐level genetic diversity (Sakai et al., 2001; Tsutsui, Suarez, Holway, & Case, 2000; Ye et al., 2004). Williams and Fishman (2014) proposed that the phenotypic divergence between introduced and native‐range populations of *Cynoglossum officinale* was mainly caused by founder effects. It was believed that small founding sizes reduced genetic variation and fitness but did not prevent adaptation if the founders originated from genetically diverse populations (Szucs, Melbourne, Tuff, Weiss‐Lehman, & Hufbauer, 2017).

In this study, we found that total biomass and height of plants from the invasive range were larger than that from the native range at high nutrient supply, but not at low nutrient level. A similar trend was proposed for the invasive plant *Poa annua*, which exerted a competitive effect on the native plant *Deschampsia*, but only at high N availability (Cavieres, Sanhueza, Torres‐Mellado, & Casanova‐Katny, 2018). Liu, Zhang, van Kleunen (2018) and Witkowski (1991) found that the increase in biomass in response to nutrient addition for invasive species is higher than for noninvasive species. At low nutrient levels, soil nutrient is a limiting factor for plant growth and may offset the competitive advantage of invasive species. Competition‐driven decreases in total biomass and plant height were significantly greater for *C. odorata* plants from the putative source populations than for those from the invasive ranges, indicating that evolution actually occurred during the invasion process of *C. odorata*.

Invasive populations of *C. odorata* are not completely released from enemies. There are more than 200 herbivores in native ranges of *C. odorata*, and 25% of them are specialists (Zhang & Feng, 2007), whereas in the species’ invasive range in China, few generalists and no specialists on *C. odorata* have been found (Xu, Xiang, Chen, & Peng, 2011). Evolution occurred in *C. odorata* plants by increasing biomass, while it also increased the secondary chemical production in response to generalists in introduced ranges. Previous studies also reported the production of higher amounts of odoratin (Eupatorium; considered as qualitative defensive compounds) in introduced *C. odorata* than in native *C. odorata* (Zheng et al., 2015). Moreover, in the plant species *Triadica sebifera*, trading off chemical defenses production occurred as a response to a coevolution with novel natural enemies in introduced ranges; this contributed to its successful invasion by enhancing competitive ability (Wang et al., 2012). Consistent with our result, Ridenour, Vivanco, Feng, Horiuchi, and Callaway (2008) proposed that “evolution occurs at increasing competitive ability and defense traits of *Centaurea maculosa* in introduced range North American.”

The secondary chemical productions quantified in this study were all flavonoids. Flavonoids are beneficial for the plant itself as physiologically active compounds, stress protecting agents, attractants, or feeding deterrents and, in general, they play a significant role in plant resistance. Acutellerin‐4`,6,7‐trimethy ether and 4`,5,6,7‐tetramethoxyflavone have defense capacity and allelopathic effect, and the concentration of these compounds in introduced *C. odorata* was significantly higher than that in putative source populations. These results suggested that evolution indeed occurred in increasing production of qualitative defensive compounds in the process of invasion of *C. odorata*. Zheng et al. (2015) considered odoratin (Eupatorium) as an important qualitative defensive compound that contributes to the successful invasion of *C. odorata*. Invasive species could enhance their competitive ability through generating many types of compounds which may only have defending herbivores ability or allelopathic effect. In our study, the other two types of extracted compounds—with high defending capacity but low allelopathy (*Isosakuranetin* and 3,5‐dihydroxy‐7,4`‐dimethoxyflavone) and low defending capacity but high allelopathy (dihydrokaempferol‐3‐methoxy ether and Kaempferide‐4`‐methoxy ether)—also contributed to the plant's strong competition ability.

Although some studies attribute the invasion success of exotic species to founder effects or biased introduction (Williams & Fishman, 2014; Yu et al., 2014), evolution in competitive plant traits indeed exists in the process of invasion (Felker‐Quinn et al., 2013). Our results indicated that *C. odorata* increased resource investment into growth through postintroduction evolution, providing more convincing evidence for the EICA hypothesis than for the use of biogeographical comparisons of invasive plants without considering their source populations. Innate competitive advantages may have contributed to the successful introduction of *C. odorata* in Asia (Qin et al., 2013), but postintroduction evolution is also essential for the species’ establishment and expanding in introduced ranges.

## AUTHORS’ CONTRIBUTIONS

This project was conceived by WTL under the supervision of YLF. YLZ, YBL, YPL, and ZYL collected the plant seeds; WTL and LKZ completed the experiments and analyzed the data. All of the authors contributed to the writing of the manuscript and approved this manuscript for submission.

## Data Availability

The data generated during this study are available from Weitao Li on reasonable request.
