# Supplementary figures and images for: Postintroduction evolution contributes to the successful invasion of Chromolaena odorata
Source: Ecol Evol. 2020 Jan 14;10(3):1252–63. doi: 10.1002/ece3.5979 (PMC7029091; doi:10.1002/ece3.5979)

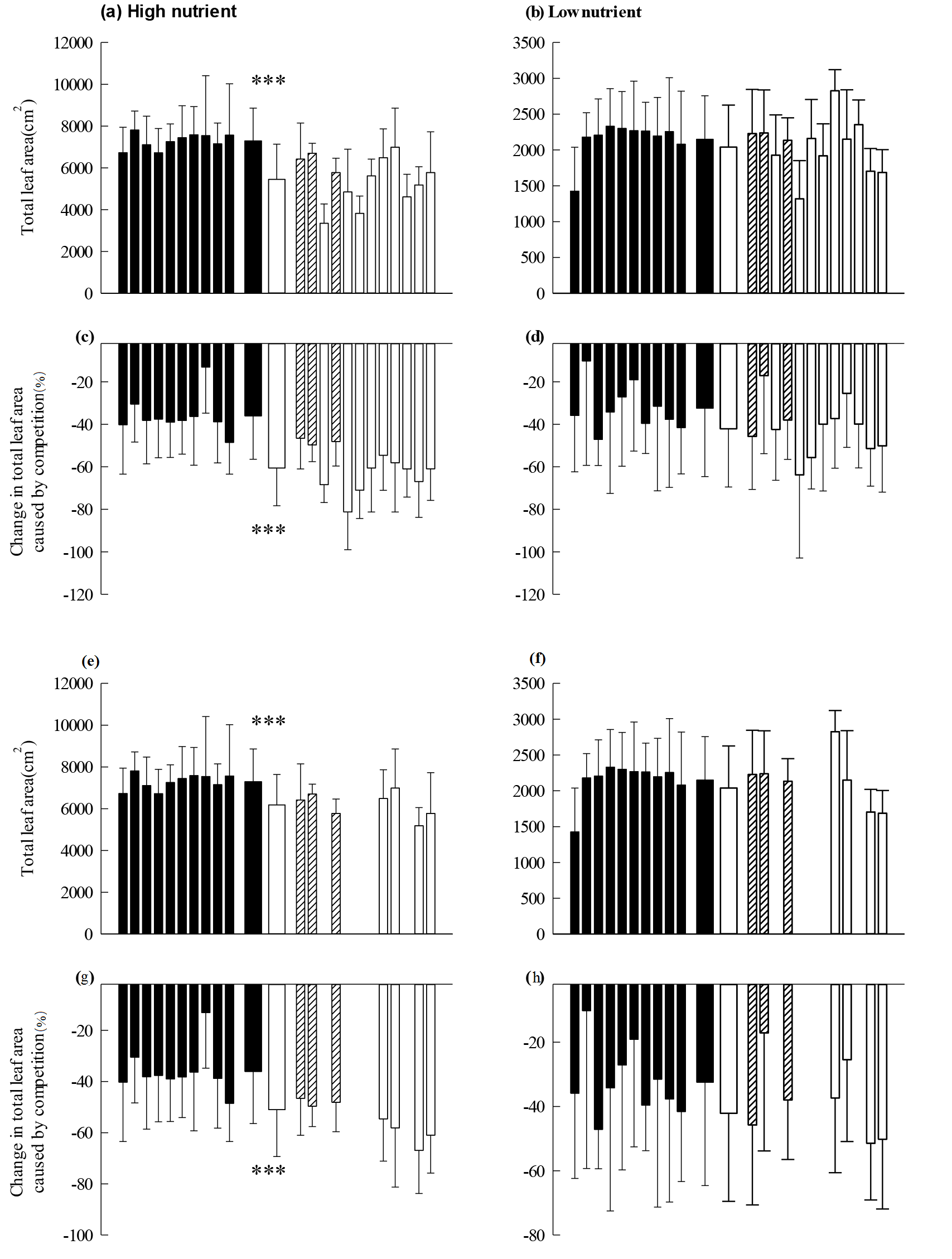

Supplement: Supplementary file 1 [file ECE3-10-1252-s001.tif]

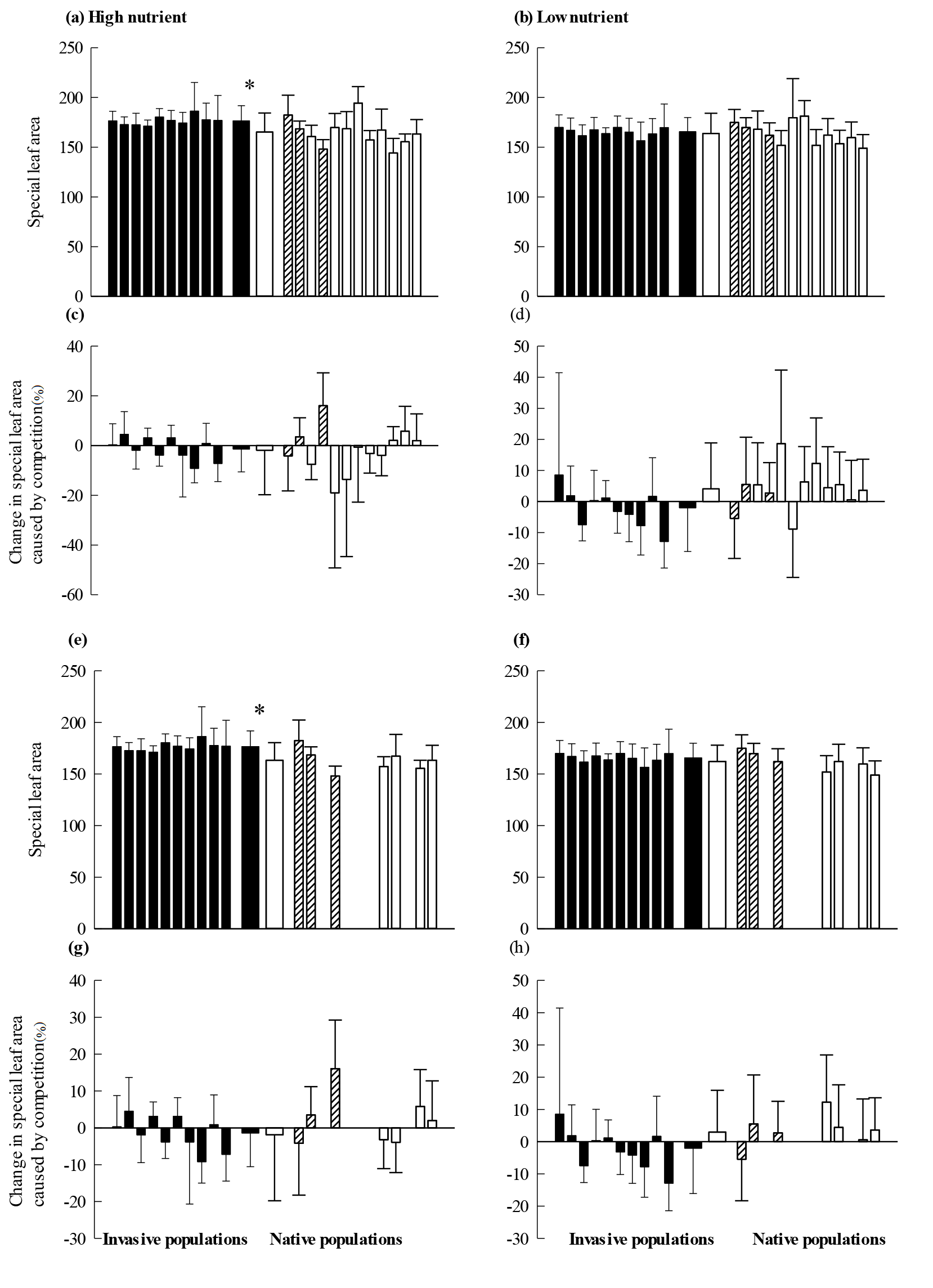

Supplement: Supplementary file 2 [file ECE3-10-1252-s002.tif]
