# Supplementary material for: Postintroduction evolution contributes to the successful invasion of Chromolaena odorata
Source: Ecol Evol. 2020 Jan 14;10(3):1252–63. doi: 10.1002/ece3.5979 (PMC7029091; doi:10.1002/ece3.5979)
Supplement: Supplementary file 3 [file ECE3-10-1252-s003.docx]

*Appendix A. Supplementary material*

Table S1 Information on 11 pairs of SSR primer for *Chromolaena odorata* (Yu and Li 2011)

| Locus Primer sequence（5′- 3′） Repeat Fluorescent No. of GenBank  motif dye used *T*_a_(°C) cycles Size range(bp) Accession No. |
| --- |
| CO15 F: GTGGGTGTTACCTCTTGG (TG)_7_ TAMRA 64 38 184-200 HM137035  R: ATCGGACATAATTTCTCCTC |
| CO26 F: CAGACTGGATCATAAGAA (TG)_8_… (TG)_3_ 58 40 265-271 HM137036  R: TTACGTGTAATAGAGCCT TAMRA |
| CO50 F: TACCCTGTTATTCCCACT (TG)_10_ FAM 60 40 283 – 311 HM137037  R: CCTAAGCCTTCTTATTTGAT |
| CO56 F: ACTGGTTGGGTTTCAATG (GT)_7_ HEX 58 38 128 – 132 HM137038  R: CTGCGTATAAAAGCGAAT |
| CO65 F: CAGTTATCTTCAACACCCAA (CT)_7_… (CT)_4_…(TC)_3_ 58 38 266 – 290 HM137039  R: TTTCCGACTAAACCCATC TAMRA |
| CO77 F: TTACCGAACGTATGTTAC (TG)_10_ HEX 50 40 141 – 143 HQ913181  R: ATGCTTCATTCTTATCCC |
| CO115 F: TCGTGGTAGAGCAGAAGA (AG)_6_GTT(AG)_4_ FAM 54 38 312 – 346 HM137040  R: AACTGCCAGATCAGGTTG |
| CO189 F: AGAGTAAGCACGAGACCG (TTTTG)_3_… (AG)_9_ 60 38 159 – 173 HM137042  R: AGAACTTTACCTCCCACA FAM |
| CO195 F: AAGAATGCACAAAATCAG (GATT)_3_… (TG)_8_ HEX 56 40 183 – 187 HM137043  R: CTTTCAGTCTCAGACGAA |
| CO227 F: GTTCGTCACCCTTTTCTC (GA)_5_… (AG)_9_ HEX 62 40 193 – 219 HM137044  R: ATCTGCACTTCATCTTCTTC |
| CO250 F: AAGGACCTCTACCTATCA (CA)_15_ FAM 58 40 79 – 95 HQ913184  R: ATTTCTGCCCATCTTATT |

Table S2. The paremeter of multiple reaction monitoring

| Compound | Parent/Daughter Ion | Cone Voltage | | Collision Energy | Dwell  Time (secs) | Ionization mode |
| --- | --- | --- | --- | --- | --- | --- |
| Isosakurnaetni | 284.86 / 163.86 | | 2.0 | 28.0 | 0.003 | ESI^-^ |
|  | 284.86 / 242.89 | | 2.0 | 19.0 | 0.003 | ESI^-^ |
| 3,5-dihydroxy-7,4`-dimethoxyflavone | 337.03 / 322.00 | | 54.0 | 16.0 | 0.003 | ESI^+^ |
| Kaempferide-4`-methoxy | 298.97 / 150.95 | | 2.0 | 28.0 | 0.003 | ESI^-^ |
| ether | 298.97 / 283.99 | | 2.0 | 22.0 | 0.003 | ESI^-^ |
| 4`, 5, 6, 7-tetramethoxy | 343.10 / 282.08 | | 82.0 | 24.0 | 0.003 | ESI^+^ |
| flavone | 343.10 / 313.11 | | 82.0 | 28.0 | 0.003 | ESI^+^ |
| Dihydrokaempferol-3-me | 300.97 / 178.98 | | 6.0 | 20.0 | 0.003 | ESI^-^ |
| thoxy ether | 300.97 / 273.04 | | 6.0 | 12.0 | 0.003 | ESI^-^ |
| Acutellerin -4`, 6, | 351.03 / 318.12 | | 46.0 | 26.0 | 0.003 | ESI^+^ |
| 7-trimethy ether | 351.03 / 336.07 | | 46.0 | 18.0 | 0.003 | ESI^+^ |
